# The Value of Cleaning a Skin Ulcer Before Obtaining a Swab to Detect *Staphylococcus aureus*, MRSA and *Streptococcus pyogenes*

**DOI:** 10.1111/iwj.71067

**Published:** 2026-10-21

**Authors:** Ulrich Orda, Sabine Orda, Ronny Gunnarsson

**Affiliations:** ^1^ Darling Downs Hospital and Health Service, Southern Cluster Warwick Hospital Warwick Queensland Australia; ^2^ School of Public Health and Community Medicine, Institute of Medicine, Sahlgrenska Academy University of Gothenburg Gothenburg Sweden; ^3^ Research, Education, Development & Innovation, Primary Health Care, Region Västra Götaland Gothenburg Sweden; ^4^ The Centre for Antibiotic Resistance Research (CARe) University of Gothenburg Gothenburg Sweden; ^5^ The College of Medicine and Dentistry James Cook University Cairns Australia

**Keywords:** infectious skin diseases, methicillin‐resistant *Staphylococcus aureus*, nucleic acid amplification techniques, skin ulcer, *Staphylococcus aureus*, *Streptococcus pyogenes*

## Abstract

Accurate identification of pathogens in clinically infected skin ulcers is essential for targeted antimicrobial therapy. Although expert consensus recommends mechanical cleansing of the ulcer bed prior to swabbing, there is a lack of empirical evidence to support this practice. This study investigated the impact of mechanical cleansing with sterile cotton gauze pads saturated with isotonic saline or clean water on microbiological results. Triple swabs were collected from 106 patients with skin ulcers at two distinct time points: prior to cleansing (baseline) and immediately following mechanical cleansing. All specimens were analysed using both conventional culture and point‐of‐care nucleic acid amplification tests (NAAT) for *Staphylococcus aureus* , methicillin‐resistant *S. aureus* (MRSA), and *Streptococcus pyogenes* . *S. aureus* was the most prevalent isolate, with detection rates of 23%–24% by culture and 44%–45% by NAAT. The prevalence and specific bacterial findings remained highly consistent before and after mechanical cleansing, with high levels of concordance across both diagnostic modalities. A sensitivity analysis excluding patients with recent antibiotic use confirmed these findings. Our results demonstrate that mechanical cleansing of the ulcer does not significantly alter the microbiological yield or improve the detection of key pathogens. These findings suggest that the traditional requirement for pre‐sampling cleansing may be unnecessary for routine diagnostic swabbing.

## Key Points

- Empirical evidence supporting the expert consensus for mechanical cleansing prior to swabbing a skin ulcer is lacking.
- In a study of 106 patients, microbiological yields evaluated via conventional culture and point‐of‐care nucleic acid amplification tests (NAAT) showed high concordance of outcome before and immediately after mechanical cleansing.
- Mechanical cleansing of the ulcer bed with sterile saline or water does not significantly improve the detection of Staphylococcus aureus, MRSA, and Streptococcus pyogenes.
- These findings suggest that the traditional requirement for mechanical cleansing before diagnostic swabbing may be unnecessary, potentially simplifying clinical workflows without compromising diagnostic accuracy.

## 1 Introduction

Bacterial skin and soft tissue infections (SSTIs) are highly prevalent, disproportionately affecting children in marginalised communities [1]. Furthermore, the global burden of these infections strongly correlates with lower socioeconomic status [2].

All open wounds rapidly become colonised by environmental and endogenous flora. The progression from microbial colonisation to subclinical (covert) infection and, ultimately, overt clinical infection exists along a continuum [3]. A positive ulcer swab identifies surface bacteria but does not inherently indicate infection. Some experts advise against microbiological analysis when an ulcer appears merely colonised or exhibits only subclinical signs of infection [3]. However, in certain patients, the clinical distinction between colonisation, clinically relevant covert infection, and overt infection can be difficult [4, 5]. Ultimately, the decision to treat is a clinical decision, ideally guided by microbiological testing.

Accurate identification of clinically significant pathogens in skin ulcers is essential for guiding targeted antimicrobial therapy. While tissue biopsy is frequently regarded as the diagnostic gold standard, superficial swabbing is generally considered sufficient to obtain a representative sample of the ulcer microbiology in most clinical scenarios [5, 6].

Current expert consensus suggests that ulcers with suspected infection should be mechanically cleansed prior to microbiological sampling [3]. However, despite the intuitive nature of this practice, the scientific evidence supporting it remains sparse. A frequently cited study by Hansson and Faegermann [7] evaluated the effects of four different antiseptic solutions on 45 venous leg ulcers; however, this research primarily assessed antiseptic efficacy rather than mechanical cleansing. To date, the only investigation comparing microbiological yields before and after mechanical cleansing with an antiseptic solution was limited to a small cohort of 11 patients [8]. Notably, no study has yet explored the impact of mechanical cleansing without using antiseptic solutions on microbiological findings.

Mechanical cleansing serves either as a primary therapeutic modality or to facilitate pathogen isolation for directed antimicrobial therapy. An objective of this study was to investigate the influence of mechanical cleansing with isotonic saline or clean water to facilitate the recovery of *Staphylococcus aureus* , MRSA, and *Streptococcus pyogenes* obtained from skin ulcers. Furthermore, we sought to determine whether the impact of cleansing differs when samples are analysed using conventional culture compared to Nucleic Acid Amplification Tests (NAAT).

## 2 Materials and Methods

### 2.1 Study Design and Setting

This prospective observational study was conducted at the emergency department (ED) of Mount Isa Hospital, a regional facility in the dry tropics of Queensland, Australia. The study was embedded within the broader ‘Prevalence of skin colonisation or infections with Methicillin‐Resistant *Staphylococcus aureus* (MRSA)/ *Staphylococcus aureus* (SA) and Group A Streptococci (GAS) in tropical Australia (SCIMAG)’ project.

### 2.2 Participant Enrolment

On 36 days between 14 May 2018 and 18 April 2019, patients in the ED waiting area were screened for eligibility between 08:00 and 24:00. All patients, regardless of their primary reason for presentation, were invited to participate. Written informed consent was obtained from all adult participants; for minors (< 16 years), consent was provided by a parent or legal guardian.

### 2.3 Clinical Examination and Sampling Protocol

Following enrolment, participants underwent a physical examination of the trunk and extremities to identify skin ulcers, while ensuring patient modesty was maintained. When an ulcer was identified, a standardised sampling protocol was followed. Triple swabs were collected using a rotational movement from the area appearing most clinically infected, similar to Levine's technique, at two distinct time points: (1) Prior to cleansing (baseline) and (2) Following mechanical cleansing of the ulcer. Physical examination and obtaining swabs were made by physicians in the emergency department volunteering to participate in the study. To minimise sequence bias during sample collection, we utilised three distinct forms, each specifying a different order for collecting the three swabs.

### 2.4 Mechanical Cleaning of the Skin Ulcer

Mechanical cleaning of the ulcer was made by physicians in the emergency department volunteering to participate in the study. Cutaneous ulcers were subjected to mechanical cleansing using a pack of three 7.5 × 7.5 cm sterile cotton gauze pads moistened with 20 mL of an isotonic saline solution; in the absence of saline, clean water was employed as a substitute. The ulcer was gently wiped twice using the gauze pads. If the ulcer was large and appeared infected, the most infected parts were cleaned. In case of no signs of infection, the whole ulcer was cleaned.

### 2.5 Microbiological Analysis

One swab of each set was placed in transport tubes with clear gel medium and dispatched daily via air freight to the Queensland Pathology laboratory in either Brisbane or Townsville. Queensland Pathology is the statewide Government pathology service. Conventional culture was performed to the same standard as for patient care using blood agar, CNA agar, and chromogenic agar specifically for MRSA identification. Transport times ranged from 4 to 29 h.

Point‐of‐care testing was performed on‐site using the remaining two swabs. One swab was analysed for *Streptococcus pyogenes* using the ID Now (Abbott) isothermal NAAT platform. Another swab was processed via the GeneXpert Xpress MRSA/SA SSTI (GXMRSA/SA‐SSTI‐10) System (Cepheid AB, Solna, Sweden) for the detection of *S. aureus* and MRSA.

A standard cotton tipped swab was used for conventional culture. For the ID Now platform the specific swab provided by ABBOTT with each test kit was used. For the Cepheid Xpert platform the specimen collection device was provided by Cepheid (P/N 900‐0370).

### 2.6 Statistical Analysis

The prevalence of skin ulcers and the detection rates for *S. aureus* , MRSA, and *S. pyogenes* were estimated. The primary outcome was the level of agreement between microbiological findings obtained before and after mechanical cleansing, which was evaluated using Cohen's kappa coefficient.

## 3 Results

### 3.1 Participation and Baseline Characteristics

A total of 791 patients were invited to participate in the study, of whom 768 (97%) provided informed consent. Skin ulcers were identified in 106 patients; the mean age of this sub‐group was 34 years (Table 1).

**TABLE 1:** Demographic information of included patients with an ulcer.

	*n* = 106
Male sex: *n* (%)	59 (56%)
Age: *n*	106
Mean age (standard deviation)	34 (22)
Median age (interquartile range)	31 (18–49)
Ethnicity	
Caucasian: *n* (%)	53 (50%)
Aboriginal and/or Torres Strait Islander: *n* (%)	43 (41%)
Other: *n* (%)	10 (9.4%)
Taken antibiotics previous week: *n* (%)	13 (12%)

### 3.2 Microbiological Prevalence

*S. aureus* was the most frequently isolated pathogen. The prevalence of *S. aureus* was 23%–24% when identified by conventional culture and 44%–45% when using NAAT platforms (Table 2).

**TABLE 2:** Bacterial findings from a skin ulcer.

	Before cleaning		After cleaning	
	Culture	NAAT	Culture	NAAT
*S. aureus*				
Scant	12		9	
Sparse	4		5	
Moderate	5		6	
Abundant	4		4	
Any presence	24% (25/104)	45% (47/105)	23% (24/105)	44% (46/105)
MRSA				
Scant	3		3	
Sparse	4		4	
Moderate	0		0	
Abundant	3		3	
Any presence	9.6% (10/104)	12% (13/105)	9.5% (10/105)	16% (17/105)
*S. pyogenes*				
Scant	3		3	
Sparse	1		3	
Moderate	6		3	
Abundant	2		3	
Any presence	12% (12/100)	18% (19/105)	11% (12/105)	19% (20/104)

### 3.3 Impact of Mechanical Cleansing on Microbiological Yield

The prevalence of *S. aureus* , MRSA, and *S. pyogenes* was similar before and after mechanical cleansing (Table 2). Comparison of the matched‐pair samples demonstrated a high level of concordance, with the majority of ulcers yielding identical microbiological results regardless of cleansing status (Table 3). Only a few cases showed a shift in status (from undetected to detected, or vice versa) following mechanical cleansing.

**TABLE 3:** Agreement between findings before and after cleaning of a skin ulcer (*n* = 106).

	*n*	Same before and after		Change after wash		Unchanged %	Cohens kappa (*p*‐value)
		Before−	Before+	Before+	Before−		
		After−	After+	After−	After+		
*S. aureus*							
Culture	104	73	18	7	6	88%	0.65 (2.7 × 10^−11^)
NAAT	105	52	40	7	6	88%	0.75 (1.6 × 10^−14^)
MRSA							
Culture	104	94	10	0	0	100%	1.0 (2.0 × 10^−24^)
NAAT	105	87	12	1	5	94%	0.77 (1.7 × 10^−15^)
*S. pyogenes*							
Culture	100	86	10	2	2	96%	0.81 (5.2 × 10^−16^)
NAAT	104	83	17	1	3	96%	0.87 (5.4 × 10^−19^)

*Note:* Including all patients with a skin ulcer.

### 3.4 Sensitivity Analysis

To evaluate the potential confounding effect of recent antimicrobial use, a sensitivity analysis was performed excluding the 13 patients who had taken antibiotics during the week preceding the study. The results remained consistent with the primary analysis, showing no significant impact of mechanical cleansing on the microbiological findings in this sub‐group (Table 4).

**TABLE 4:** Agreement between findings before and after cleaning of a skin ulcer (*n* = 93).

	*n*	Same before and after		Change after wash		Unchanged %	Cohens kappa (*p*‐value)
		Before−	Before+	Before+	Before−		
		After−	After+	After−	After+		
*S. aureus*							
Culture	91	63	15	7	6	86%	0.60 (8.1 × 10^−9^)
NAAT	92	47	35	6	4	89%	0.78 (7.5 × 10^−14^)
MRSA							
Culture	91	83	8	0	0	100%	1.0 (1.4 × 10^−21^)
NAAT	92	79	10	1	2	97%	0.85 (3.0 × 10^−16^)
*S. pyogenes*							
Culture	87	75	9	2	1	97%	0.84 (5.1 × 10^−15^)
NAAT	91	73	15	0	3	97%	0.89 (1.4 × 10^−17^)

*Note:* Only including patients with a skin ulcer that has not taken antibiotics the preceding week.

## 4 Discussion

The primary finding of this study is that mechanical cleansing of a skin ulcer with clean water or isotonic saline does not significantly alter the recovery of potentially pathogenic bacteria. This lack of a clinically relevant effect was consistent across both conventional culture and NAAT‐based molecular analyses.

### 4.1 The Clinical Significance of Pre‐Sampling Cleansing

Mechanical cleansing prior to obtaining a microbiological swab has long been recommended based on expert consensus and traditional clinical practice [9]. However, empirical evidence to support this practice is remarkably sparse. Ernlund [8] compared swabs before and after cleansing venous stasis ulcers in a small cohort of 11 patients using hypochlorous acid or chlorhexidine. While the bacterial species identified remained consistent, a reduction in the abundance of growth was noted post‐cleansing. Similarly, Hansson [7] mentioned a 10‐patient pilot study in which samples were taken before and after cleansing. Based on those pilot results—which were not formally presented—they proceeded to sample the remaining 48 patients without prior cleansing.

Apart from these limited investigations, which featured small sample sizes and partially undisclosed data, there is a lack of published research exploring the impact of mechanical cleansing on microbiological yields. Our study represents the first substantial investigation into this topic. Our results suggest no clear diagnostic benefit to mechanical cleansing of the ulcer prior to swabbing, regardless of whether isothermal or thermal‐cycling NAAT or conventional culture is employed.

### 4.2 Strengths and Limitations

A major strength of this investigation is the head‐to‐head comparison of cleansing effects using both NAAT and conventional culture within a clinical setting. Although this study utilised a convenience sample rather than a population‐based cohort, the observed prevalence of skin ulcers and the identified pathogens are consistent with previous epidemiological data. Therefore, there is little reason to suspect that the impact of ulcer cleansing would differ significantly in other clinical contexts.

While a strictly standardised swabbing protocol performed by a single investigator would have minimised sampling variation, we opted to have multiple emergency department physicians voluntarily participating to collect swabs. Although this pragmatic approach introduces minor variations in swabbing technique, it more accurately reflects real‐world clinical practice. Consequently, this methodological choice serves as both a limitation in standardisation and a strength in ecological validity.

Furthermore, antiseptic solutions were intentionally excluded to isolate the specific effects of mechanical cleansing. Utilising antiseptics would have introduced a confounding variable, making it difficult to distinguish whether outcomes were driven by mechanical action or the solution's antimicrobial properties. This design choice does not discount the clinical efficacy of antiseptics, but rather reflects the specific focus of this study.

## 5 Conclusions

Despite the prevailing clinical perception that mechanical cleansing improves the accuracy of microbiological sampling, our findings demonstrate that it does not significantly improve the detection of *S. aureus* , MRSA, or *S. pyogenes* . This study suggests that the traditional requirement for cleansing may be unnecessary for routine diagnostic swabbing.

## Funding

This research was supported by the Emergency Medicine Foundation (EMF; grant number EMSS‐311R26‐2016‐ROFE) and the Western Queensland Primary Health Network (WQPHN), which provided funding for the NAAT platforms and associated test kits. The authors, alongside the participating medical staff of the emergency department, contributed their professional time to the study without external financial remuneration.

## Ethics Statement

The study protocol was formally approved by the Townsville Human Research Ethics Committee (HREC/17/QTHS/158). All research procedures were conducted in strict accordance with the ethical principles of the Declaration of Helsinki. Written informed consent was obtained from all participants or their legal guardians prior to enrolment.

## Conflicts of Interest

The authors declare no conflicts of interest.

## Data Availability

The data that support the findings of this study are available on request from the corresponding author. The data are not publicly available due to privacy or ethical restrictions.
